# The distribution and maturation of tertiary lymphoid structures can predict clinical outcomes of patients with gastric adenocarcinoma

**DOI:** 10.3389/fimmu.2024.1396808

**Published:** 2024-07-29

**Authors:** Hui Sun, Yuxi Liu, Wanjing Cheng, Rong Xiong, Wenchao Gu, Xiaoyan Zhang, Xin Wang, Xu Wang, Cong Tan, Weiwei Weng, Meng Zhang, Shujuan Ni, Dan Huang, Midie Xu, Weiqi Sheng, Lei Wang

**Affiliations:** ^1^ Department of Pathology, Fudan University Shanghai Cancer Center, Shanghai, China; ^2^ Department of Oncology, Shanghai Medical College, Fudan University, Shanghai, China; ^3^ Institute of Pathology, Fudan University, Shanghai, China; ^4^ Pathology Department of HaiNan General Hospital, HaiNan, China; ^5^ Department of Diagnostic and Interventional Radiology, University of Tsukuba, Tsukuba, Japan

**Keywords:** stomach adenocarcinoma, tertiary lymphoid structure (TLS), distribution of TLSs, maturation of TLSs, DC-LAMP+ dendritic cells, prognosis

## Abstract

**Introduction:**

Tertiary lymphoid structures (TLSs) are analogues of secondary lymphoid organs that contain various immune cells. The spatial distribution, maturation and composition of TLSs have differential effects on prognosis, and the roles of TLSs in gastric adenocarcinoma (GA) have not been revealed.

**Methods:**

Thus, we evaluated the prognostic value of TLSs in GA through analysis of bulk RNA sequencing(RNA-seq) data from public databases and validated our findings in tumour samples from the Fudan University Shanghai Cancer Center (FUSCC) cohort. The spatial distribution,maturation, and composition of TLSs in GA were analysed by reviewing H&E-stained sections and by multiplex immunofluorescence (mIF) staining.

**Results:**

We found that TLSs, especially TLSs with germinal centres (GCs) and TLSs located in the invasive margin (IM), were correlated with prolonged overall survival (OS). Second, analysis of public RNA-seq data showed that high dendritic cell (DC) scores were a favourable prognostic factor in GA patients with high scores for both TLSs and GCs. In the FUSCC cohort, DC-LAMP+ DCs weresignificantly enriched in IM-TLSs with GCs, suggesting a potential correlation between the tumour immune activation milieu and the DC abundance. Third, compared to that in TLSs without GCs, the proportion of FOXP3+CD8+ Treg cells was significantly decreased in IM-TLSs with GCs, and the percentage of PD1+CD20+ B cells was significantly increased in TLSs with GCs.

**Discussion:**

Our results demonstrate that the spatial arrangement and maturation of TLSs significantly affect prognosis and indicate that TLSs could be a new additional factor for histopathological evaluation.

## Introduction

Gastric adenocarcinoma (GA) is the fifth most frequently diagnosed cancer and the second leading cause of cancer death worldwide, responsible for more than 1 million new cases and approximately 770 000 deaths in 2020, with approximately half (478 000) of the new cases diagnosed in China (1). The prognosis of advanced GA, a heterogeneous disease, remains poor due to the lack of effective therapies; thus, personalised treatment approaches are needed (2). With the application and development of immunotherapy, scientists have discovered that the immune microenvironment affects not only the efficacy of immunotherapy but also the biological behaviour of the tumour cells within it (3). Extensive molecular studies have attempted to identify GA subtypes, revealing a crucial role of the immune microenvironment in the progression of GA (3). Zhang et al. demonstrated that large numbers of tumour-infiltrating lymphocytes (TILs) were associated with a favourable prognosis in patients with GA and that the presence of TILs indicates a protective host antitumour immune response (4). Therefore, direct characterisation of the immune context may provide further insights into this malignancy.

Recently, tumour-associated tertiary lymphoid structures (TLSs), which are analogues of secondary lymphoid organs, have attracted extensive attention (5). These complex structures constitute privileged sites for local antigen presentation and lymphocyte differentiation, thus providing an important milieu for both cellular and humoral antitumour immune responses (6–8). Indeed, the presence of tumour-associated TLSs is associated with a favourable prognosis and a robust response to immunotherapy in most solid tumours, albeit with some contradictory observations. Further studies showed that both the total number of TLSs and the numbers of TLS-associated immune cells – including but not limited to follicular helper T cells (TFHs), follicular B cells, mature DCs, and CD8+ T cells – and HEVs were associated with prolonged survival in patients with many different tumour types (9–13). Regarding maturity, the first prognostic analysis of TLSs in human cancer patients revealed that TLSs containing GCs were correlated with improved survival in patients with hepatocellular carcinoma (HCC)and colorectal cancer (CRC) (14, 15). However, studies of TLSs in GA have generally focussed on B cells and CD8+ T cells (16–18). The maturation of TLSs in GA has been investigated only in research on Epstein−Barr virus-associated gastric cancer (EBVaGC). This study revealed that intratumoural mature TLSs were associated with a favourable prognosis and predicted a good therapeutic response in EBVaGC patients (19). However, the prevalence of EBVaGC in China was estimated to be only 4.1% (55 of 1328 cases) (20), and the prognostic value of TLS maturation in conventional GA remains unknown. Moreover, whether immune components within TLSs impact the maturation of TLSs in GA awaits further exploration. Ding et al. demonstrated that the abundance of intratumoural TLSs was an effective predictor of a favourable prognosis for patients with intrahepatic cholangiocarcinoma (iCCA), while the presence of peritumoural TLSs was significantly associated with dismal outcomes (21). The dual impacts of spatially different TLS distributions have also been recognised in HCC and breast cancer (5, 22). However, the impact of the spatial distribution of TLSs in GAs remain inconclusive.

Recently, much effort has been directed at studying the role of TLSs in tumours using RNA-seq data, and considerable progress has been made. Jia et al. divided the TCGA-Liver Hepatocellular Carcinoma cohort into intratumoural TLS-positive (iTLS-positive) and intratumoural TLS-negative (iTLS-negative) groups using pathological sections from the Cancer Digital Slide Archive, and they discovered from the RNA-seq data that the differentially expressed genes between the two groups were closely associated with immune-dominated pathways (23). Via RNA-seq analysis of pancreatic ductal adenocarcinoma samples, Gunderson et al. discovered that the differentially expressed genes in the TLS-positive (TLS+) and TLS-negative (TLS-) subtypes were associated with T and B-cell activity, and gene set enrichment analysis (GSEA) revealed significant upregulation of MYC signalling and interferon-alpha signalling in TLS+ tumours (24). Along with studies investigating TLSs by RNA-seq analysis, studies using public data to quantify and analyse TLSs have also been conducted. Gene signatures representing TLSs have been reported. Coppola et al. discovered a strong correlation between a 12-chemokine gene signature and the presence of ectopic lymph node-like structures that were associated with better patient survival outcomes (25). In addition to TLSs, GC-related gene signatures have also been employed in TLS-related studies. For example, He et al. used GC markers to compute GC scores in single-cell RNA sequencing (scRNA-seq) samples and discovered that in non-small cell lung cancer, while GC+ TLSs were associated with a significantly lower risk of recurrence, GC− TLSs showed no prognostic significance (26).

Here, we comprehensively evaluated the GA H&E-stained sections that TLSs at different stages of maturation and with different spatial distributions exhibit varying prognostic value; particularly, TLSs with GCs and TLSs located in the invasive margin (IM) were identified as crucial prognostic indicators in GA. The cellular composition of TLSs was evaluated by multiplex immunofluorescence (mIF) staining, which revealed that the frequency of DCs in TLSs with GCs and located in the IM was significantly increased. Together with the findings from analysis of public RNA-seq data showing that the DC score was a favourable prognostic factor in patients with GA with high scores for both TLSs and GCs, we speculated that there is a potential correlation between the antitumour immune activation milieu and the abundance of mature DCs. Finally, GSEA was applied to analyse the unique pattern of gene expression in TLS+ GA samples, and pathways related to the maturation and function of DCs were found to be enriched in the TLS+ group, which again complemented the above findings.

## Materials and methods

### Public RNA-seq data collection and preprocessing

The RNA-seq data and matching clinical information of GA patients were extracted from two independent GA datasets, GSE62254 (https://www.ncbi.nlm.nih.gov/geo/query/acc.cgi?acc=GSE62254, n=300) and GSE29272 (https://www.ncbi.nlm.nih.gov/geo/query/acc.cgi?acc=GSE29272, n=126). Samples without matching survival data were excluded from the analysis. We employed a published TLS-related gene signature to represent TLSs (25). CD21, CD23 and CXCL13 were used as the signature genes to represent GCs (13). The abundances of activated DCs and other immune cells were also determined using published gene signatures (27) (**Supplementary Table 1**). After calculating the scores of each sample using the single-sample gene set enrichment analysis (ssGSEA) method, tumour samples with a low TLS score were classified as TLS-, tumour samples with a high TLS score were classified as TLS+, and classification and scoring based on GCs and on DCs and other immune cells were performed similarly.

### Tumour specimens

The discovery cohort consisted of 166 patients who had undergone surgical resection for GA at FUSCC between 2016 and 2021. The following clinical and biological features were recorded: age, sex, GA features and follow-up data (e.g., tumour size, histologic grade, depth of invasion, vascular invasion status, perineural invasion status, lymphatic metastasis status and distant metastasis status). The TNM classification system was used to evaluate the clinical stage of GA (28). None of the patients had undergone preoperative treatment, 85 patients had been treated with a postoperative combined regimen of chemotherapy and radiotherapy, and 81 patients had been treated with postoperative chemotherapy. All specimens were collected from patients who provided informed consent, and our study was approved by The Research Ethics Committee of FUSCC.

### Characterisation and quantification of TLSs

In the present study, TLS evaluation was retrospectively performed using H&E-stained sections from the FUSCC cohort. In brief, the tumour tissue sections were independently reviewed by three pathologists (Hui Sun, Wanjing Cheng, Lei Wang) who were trained on the TLS scoring system (5) and blinded to the clinical data, and TLSs were scored using a previously published scale. No uniform standard was available for the histopathological TLS scoring system, and TLSs were defined as organised lymphoid aggregates (21). In brief, TLSs were classified as follows: 1) early TLSs (E-TLSs): ill-defined clusters of lymphocytes representing the first stage of TLS development; 2) primary follicle-like TLSs (PFL-TLSs): follicular dendritic cell (FDC)-containing TLSs without GCs; and 3) secondary follicle-like TLSs (SFL-TLSs): GC-containing TLSs. We divided TLSs into two categories according to the GC status: E-TLSs and PFL-TLSs did not contain GCs, and SFL-TLSs contained GCs.

To determine the spatial distribution of TLSs, the tumour area was subdivided into the tumour centre (CT) and the IM (the 2.5 mm-wide region on each side of the intra- and extratumoural boundaries) regions according to criteria defined in previous studies (4). Next, all available complete sections were morphologically analysed for the (i) number and (ii) density of TLSs. The TLS density was calculated as the number of TLSs per mm2 in the CT and IM regions. Following the TLS assessment, a set of TLS scores was obtained that included (i) the CT-TLS number (CT-TLS-N), which indicated the number of TLSs in the CT region; (ii) the CT-TLS density (CT-TLS-D), which was calculated by dividing the CT-TLS number by the area of the CT region and reflected the distribution and density of TLSs in the CT region; (iii) the IM-TLS number (IM-TLS-N), which represented the number of TLSs in the IM region; (iv) the IM-TLS density (IM-TLS-D), which was calculated by dividing the IM-TLS number by the IM region and reflected the distribution of TLSs in the IM region; (v) the TLS-SUM number (Total-TLS-N), which indicated the total number of TLSs in both the CT and the IM regions; and (vi) the TLS-SUM density (Total-TLS-D), which was calculated by dividing the TLS-SUM number by the sum of the areas of the CT and IM regions and reflected the distribution and density of TLSs in the overall (SUM) tumour region.

### Multiplex immunofluorescence(mIF)

To investigate the cellular composition of TLSs with differences in maturation and localisation, mIF staining was conducted. For mIF staining, an anti-CD20, anti-CD8, anti-DC-LAMP, anti-CD23, anti-PD1, and anti-Foxp3 antibody panel and an Opal 7-Color Manual IHC Kit (50-slide kit, Perkin Elmer/Akoya, NEL871001KT, USA) were used. Before an experiment was started, the tissue slides were baked for 4 hours at 65°C in an oven. Primary antibodies specific for the following proteins were used: CD20 (ab78237, 1:100 dilution, Abcam, USA), CD8 (ab237709, 1:500 dilution, Abcam, USA), DC-LAMP (DDX0191P-100, 1:100 dilution, Dendritics, France), CD23 (ab92495, 1:200 dilution, Abcam, USA), PD1 (D4W2J, 1:250 dilution, Cell Signaling Technology, USA), and Foxp3 (D2W8E, 1:100 dilution, Cell Signaling Technology, USA). The slides were incubated for 60 minutes with the primary antibody and rinsed three times with TBST buffer for two minutes each prior to detection using Polymer HRP Ms+Rb for 10 minutes and incubation with Opal dye (1:75 dilution) for 10 minutes. This process was repeated for each antibody used for staining. Nuclei were subsequently visualised by detecting nuclear DNA using 4’,6-diamidino-2-phenylindole (DAPI). The sections were mounted with ProLongTMDiamond (IntrogenTM, cat p36970, Thermo Fisher Scientific Inc., USA).

### Image acquisition and quantitative analysis

All immunofluorescence-stained slides were scanned using a digital slide scanner (Pannoramic MIDI, 3DHISTECH Ltd, Hungary), and staining was then independently analysed and quantified by three experienced pathologists using HALO (v2.2.1870.17, Indica Labs, Albuquerque, NM, USA). The whole slide scan was performed at 100x magnification, and multispectral high-power fields were imaged at 200x. To acquire reliable unmixed images, library slides were created by staining a representative sample with each of the specific dyes. This spectral library served as a reference for target quantitation; the intensity of each fluorescent target was extracted from the multispectral data by linear unmixing. The software output the total number of cells and the number of, percentage, and fluorescence intensity of positive cells.

### Statistical analysis and visualisation

(https://www.r-project.org/) and GraphPad Prism version 9.0 (GraphPad Software, San Diego, CA, USA). The optimal cut-off value for continuous variables was determined using the ‘maxstat’ R package. Survival analysis was performed using the ‘survival’ R package. Survival distributions were compared using the Kaplan−Meier method and the log-rank test. Univariate and multivariate analyses were performed using a Cox proportional hazards regression model. Comparisons between two groups and among more than two groups were performed by the χ2 test, two-tailed Student’s t test, or one-way ANOVA as appropriate. The correlation between the percentage of positive cells and the fluorescence intensity of each marker in positive cells was assessed by Pearson correlation analysis. A two-sided p value < 0.05 was considered to indicate statistical significance. GSEA software was used to analyse pathway enrichment in the TLS+/- groups in both public cohorts. GSEA plots were generated and optimised using the ‘enrichplot’ package.

## Results

### Analysis of RNA-seq data suggests that TLSs and GCs are favourable prognostic factors for GA

**Figure 1:**
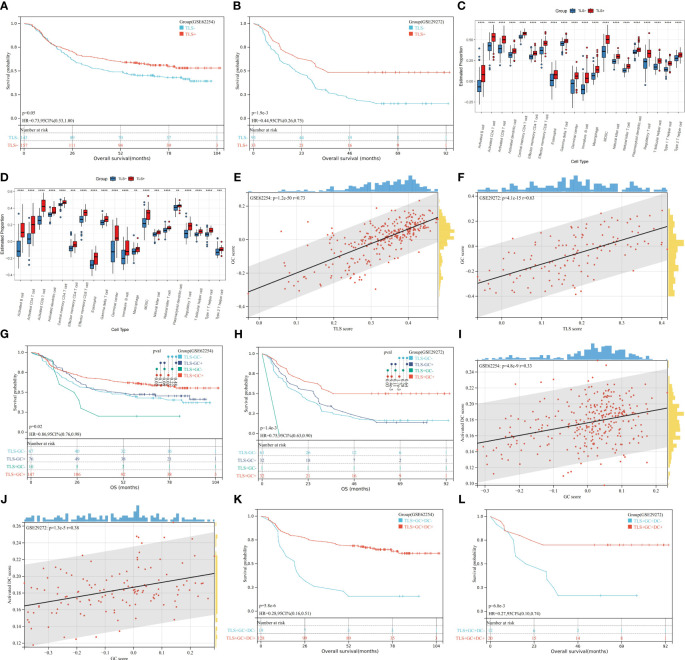
Prognostic value of TLSs, GCs, and DCs and their correlations at the transcriptome level. **(A, B)** Kaplan–Meier OS analysis with the log-rank test showed significant differences between the curves of the TLS+ group and TLS- group in the GSE62254 **(A)** and GSE29272 **(B)** datasets. **(C, D)** The numbers of GCs and infiltration of immune cells were compared between the TLS+ group and the TLS- group in the GSE62254 **(C)** and GSE29272 **(D)** datasets by the Wilcoxon rank-sum test. *p<0.05, **p<0.01, ***p<0.001, ****p<0.0001. **(E, F)** The rank correlation method was used to examine the correlation between the TLS score and the GC score in the GSE62254 **(E)** and GSE29272 **(F)** datasets; r denotes the Spearman correlation coefficient. **(G, H)** Kaplan–Meier OS analysis with the log-rank test showed significant differences among the curves of the TLS-GC- group, TLS-GC+ group, TLS+GC- group, and TLS- group in the GSE62254 **(G)** and GSE29272 **(H)** datasets. **(I, J)** The rank correlation method was used to examine the correlation between the GC score and the DC score in the GSE62254 **(I)** and GSE29272 **(J)** datasets; r denotes the Spearman correlation coefficient. **(K, L)** Kaplan–Meier OS analysis with the log-rank test showed significant differences between the curves of the TLS+GC+DC- group and the TLS+GC+DC+ group in the GSE62254 **(K)** and GSE29272 **(L)** datasets.

Public databases were analysed to determine the prognostic significance of the TLS gene signature. In both the GSE62254 and GSE29272 datasets, we found that patients with TLS+ samples had longer overall survival (OS) times than patients with TLS- samples (GSE 62254, p=0.05; GSE29272, p=1.9e-3) (**Figures 1A, B**). This finding corroborates the conventional understanding of the role of TLSs as a hub for anti-tumour immunity. As shown in **Figures 1C, D**, the TLS+ and TLS- groups were entirely different in terms of the tumour immune microenvironment (TIME), with the TLS+ group having higher scores for GCs and almost every kind of immune cell, including activated DCs. GCs are frequently observed in TLSs. Spearman correlation analysis of TLS and GC scores revealed that TLSs were strongly correlated with GCs (GSE62254, r=0.73; p=1.2e-50; GSE29272, r=0.63, p=4.1e-15) (**Figures 1E, F**). Moreover, in both datasets, the TLS+GC+ group had a better prognosis than the TLS+GC- group did (OS; GSE62254, p=0.02; GSE29272, p=0.03) (**Figures 1G, H**), a pattern that was not observed between the TLS-GC+ and TLS-GC- groups. The TLS+GC+ group was also shown to have the highest survival probability among all four groups (TLS+GC+, TLS+GC-, TLS-GC+, TLS-GC-). Collectively, these findings suggest that the presence of TLSs with GCs but not TLSs alone is a favourable prognostic factor in patients with GA. Martínez-Riaño et al. thoroughly explained how FDCs, which are located in the GC, serve as a key source of antigens for GC B cells by presenting immune complexes (29). This observation prompted us to explore the correlations among DCs, GCs and TLSs. RNA-seq data analysis demonstrated a weak correlation between the GC score and the DC score (Spearman correlation analysis; GSE62254, r=0.33, p=4.8e-9; GSE29272, r=0.38, p=1.3e-5) (**Figures 1I, J**). We also found that the TLS+GC+DC+ group had a better prognosis than the TLS+GC+DC- group (OS; GSE62254 p=5.8e-6, GSE29272 p=6.8e-3) (**Figures 1K, L**), suggesting that DCs are a prognostic factor in TLS+GC+ GA.

### Clinicopathological characteristics of patients and the development of tumour-associated TLSs through sequential stages of maturation in GA specimens

After determining the correlation between TLSs and patient prognosis via RNA-seq data analysis, we next verified these findings in paraffin-embedded sections from the FUSCC cohort.

A total of 166 patients with stage II and III GA treated with gastrectomy were included. Among the patients, 101 (61.2%) were male and 65 were female. The patients’ ages ranged from 23 to 77 years (median, 57 years; mean, 55 years). Overall, 81 patients were treated with chemotherapy, and 85 patients were treated with a combination of chemotherapy and radiotherapy after gastrectomy. None of the patients had undergone preoperative treatment.

**Figure 2:**
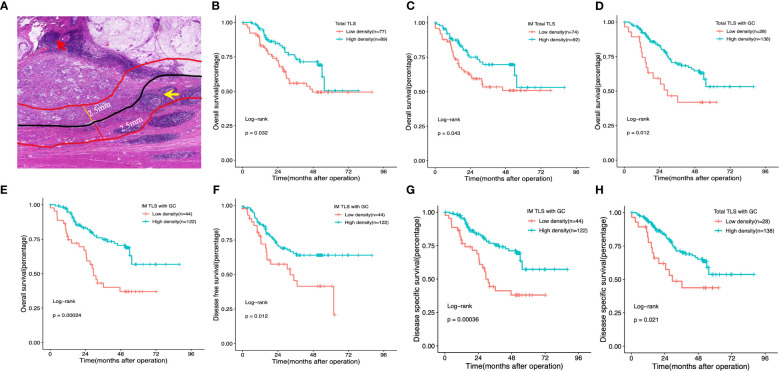
Impact of the density, maturation, and spatial distribution of tertiary lymphoid structures on patient survival outcomes. **(A)** Histological appearance of tertiary lymphoid structures associated with the invasive margin and the centre of the tumour. Red arrow: TLS in the centre of the tumour. Yellow arrow:TLS in the invasive margin of the tumour. **(B, D)** Kaplan–Meier OS analysis with the log-rank test showed significant differences between the curves of patients stratified by the density of total TLSs **(B)**, total IM-TLSs **(C)**, and total TLSs with GCs **(D)** (high- density and low- density). **(E–G)** Kaplan–Meier analysis of OS **(E)**, DFS **(F)** and DSS **(G)** with the log-rank test showed significant differences between the curves of patients stratified by the density of IM-TLSs with and without GCs (high density and low density). **(H)** Kaplan–Meier DSS analysis with the log-rank test showed significant differences between the curves of patients stratified by total TLSs with GCs. Red arrow: TLS in the centre of the tumour. Yellow arrow: TLS in the invasive margin of the tumour.

We initially assessed the presence of TLSs in H&E-stained primary GA tissues using full diagnostic assessments of 166 patients from the FUSCC. As shown in **Figure 2A**, TLSs were observed in the GA tissues, but there was a spatial distribution of TLSs in the tumour, the CT and the peritumoural area (i.e., the IM), and we evaluated the distributions of total TLSs, CT-TLSs (red arrow), and IM-TLSs (yellow arrow) separately. Database analysis revealed that patients with high scores for both TLSs and GCs had a better prognosis. We further classified TLSs into two categories according to GC status, namely, TLSs with GCs and TLSs without GCs. E-TLSs and PFL-TLSs were classified as TLSs without GCs (**Figure 2A**), and SFL-TLSs were classified as TLSs with GCs (**Figure 2A**), in which centrocytes, centroblasts, and phagocytosis by macrophages were observed.

How the spatial distribution, maturation, and composition of tumour-associated TLS in clinical tumour specimens from our centre influence the prognosis of patients with GA. With these questions in mind, we comprehensively evaluated the prognostic value, spatial distribution, maturation, and composition of TLSs in paraffin-embedded sections of GAs.

### Prognostic value of TLSs based on density, maturation, and spatial location in GA patients from FUSCC

To test the prognostic significance of the TLS density in our cohort, we defined a threshold for separating patients with high and low TLS densities with the ‘maxstat’ R package (**Supplementary Table 2**). Kaplan–Meier survival analyses revealed that a high density of total TLSs (p=0.032, **Figure 2B**) and total TLSs located in the IM (p=0.043, **Figure 2C**) were significantly correlated with prolonged OS, while total TLSs located in the CT were not correlated with prognosis. We also assessed the maturation stages of TLSs and revealed that a high density of total TLSs with GCs was significantly correlated with prolonged OS(p=0.012, **Figure 2D**) and disease-specific survival (DSS; p=0.021, **Figure 2H**), while a high density of TLSs with GCs located in the IM (IM-TLS with GCs) was significantly correlated with improved OS (p=0.00024, **Figure 2E**), DSS(p=0.00036, **Figure 2G**) and disease-free survival (DFS; p=0.012, **Figure 2F**). In addition, the associations of clinicopathological parameters with OS, DSS and DFS of FUSCC were listed in **Table 1**. Among all the significant covariates identified by univariate analysis, peritoneal metastasis and the density of IM-TLSs with GCs were found to be independent prognostic factors for both OS and DFS in multivariate Cox regression analysis. Notably, IM-TLSs with GCs had a hazard ratio (HR) of <1, suggesting that a high number of IM-TLSs with GCs had a protective effect on OS and DFS (HR = 0.41, 95% confidence interval [CI]: 0.23–0.75, p = 0.003; HR = 0.55, 95% CI: 0.30–1.00, p = 0.049].

**Table 1:** Univariate and multivariate analyses of clinicopathological factors for overall survival and disease free survival in gastric cancer patients from FUSCC (166 cases).

Variable	Overall survival				Disease specific survival				Disease free survival					
	Univariate analysis		Multivariate analysis		Univariate analysis		Multivariate analysis		Univariate analysis		Multivariate analysis			
	HR (95% CI)	p^a^	HR (95% CI)	p^a^	HR (95% CI)	p^a^	HR (95% CI)	p^a^	HR (95% CI)	p^a^	HR (95% CI)	p^a^	HR (95% CI)	p^a^
Age (≥ 55/< 55)	1.11(0.65-1.89)	0.70			1.12(0.66- 1.93)	0.67			0.81(0.48-1.35)	0.42				
Gender (Male/Female)	1.06(0.62-1.82)	0.83			1(0.58-1.73)	0.99			0.77(0.46-1.29)	0.32				
Location (Cardia/Fundus/Antr)	1.94(0.52-0.58)	0.91			1.84(0.54-0.6)	0.33			2.06(0.48-0.34)	**<0.005**	2.66(1.29-5.10)		**0.003**	
Tumour mass size (≥ 4.7/< 4.7cm)	1.78(1.05-3.00)	**0.03**	1.50(0.86-2.62)	0.157	1.78(1.04- 3.03)	**0.032**	1.21 (0.66- 2.22(	0.544	1.11(1.14-3.21)	**0.012**	1.27 (0.73-2.22)		0.395	
Lauren classification (Intesinal/Diffuse/Mixed subtype)	1.04(0.72-1.51)	0.53			1.16(0.8-1.68)	0.421			1.05(0.72-1.53)	0.620				
Histologic grade (Poor, Undifferented/ Good, mod)	1.55(0.56-4.29)	0.39			1.14(0.55-2.36)	0.727			1.58(0.57-4.37)	0.378				
Tumour depth (T3, T4/T1, T2)	3.33 (1.04-10.65)	**0.032**	1.02(0.25-4.09)	0.978	3.22(1-10.32)	**0.049**	0.94 (0.22-3.96)	0.929	3.29(1.03-10.52)	**0.034**	0.99 (0.25- 3.85)		0.987	
Vascular invasion (Present/Absent)	0.36(0.21-0.61)	**0.017**	1.38(0.50-3.83)	0.538	2.82(1.12-7.12)	**0.028**	1.58 (0.56- 4.45)	0.386	3.7(1.33-10.23)	**0.007**	2.14 (0.61-7.44)		0.233	
Nervous invasion (Present/Absent)	2.7 (1.08-6.78)	**0.027**	2.53(0.87-7.37)	0.088	2.61(1.04-6.56)	**0.041**	2.28 (0.76- 6.86)	0.143	2.13(0.92-4.97)	**0.072**	1.72 (0.61-4.89)		0.307	
Lymphatic metastasis (>=10/<10)	1.81(1.06-3.08)	**0.027**	1.17(0.63-2.20)	0.620	1.84(1.07-3.17)	**0.028**	1.11 (0.61- 2.05)	0.729	2.55(1.48-4.39)	**0.00049**	0.98(0.52-1.87)		0.962	
Peritoneal metastasis (Present/Absent)	3.35(1.92-5.87)	**0.001**	2.54(1.38-4.70)	**0.003**	3.58(2.03-6.3)	**0**	2.92(1.48- 5.76)	**0.002**	4.27(2.42-7.51)	**0.000**	7.48 (4.13-13.57)		**<0.001**	
pTNM stage (III +II+I)	4.13(1.35-12.64)	**0.021**	1.81(0.51-6.49)	0.362	4.01(1.31-12.28)	**0.015**	1.78(0.49- 6.43)	0.378	3.29(1.03-10.52)	**0.005**	1.48(0.27- 8.14)		0.653	
Treatment (ChT/CRT)	0.77(0.43-1.37)	0.37			0.76(0.42-1.37)	0.36			1.35(0.79-2.30)	0.27				
CT Total TLS (Low/High)	0.65(0.38-1.11)	0.11			1.06(0.56-2.03)	0.85			1.58(0.79-3.18)	0.19				
CT TLS with GC (Low/High)	0.63(0.33-1.19)	0.157			0.7(0.39-1.24)	0.223			0.79(0.42-1.49)	0.46				
IM Total TLS (Low/High)	0.53(0.29-0.99)	**0.044**	0.93(0.46-1.86)	0.834	0.64(0.37-1.09)	0.10			0.77(0.46-1.28)	0.312				
IM TLS with GC (Low/High)	0.39(0.23-0.65)	**<0.005**	0.41(0.23- 0.75)	**0.003**	0.39(0.23- 0.67)	**<0.001**	0.52 (0.28- 0.97)	**0.041**	0.51(0.30-0.87)	**0.012**	0.55 (0.30-1.00)		**0.049**	
Total TLS (Low/High)	0.56(0.33-0.96)	**0.035**	0.76(0.41- 1.42)	0.395	0.7(0.4- 1.22)	0.21			0.79(0.51-1.38)	0.69				
Total TLS with GC (Low/High)	0.58(0.34-0.99)	**0.044**	0.64(0.32-1.29)	0.214	0.49(0.27-0.91)	**0.024**	0.76 (0.36- 1.61)	0.473	0.53(0.30-1.00)	0.068				

HR, Hazard ratio; CI, confidence interval; ^a^All statistical tests were 2-sided. Significance level: p < 0.05. The bold values represent p<0.05.

Having determined the prognostic value of IM-TLSs with GCs in FUSCC cohort, the correlations between IM-TLSs with GCs and clinicopathological characteristics were further explored. As shown in **Table 2**, the density of IM-TLSs with GCs in GA patients was correlated with histologic grade (P=0.0001), depth of tumour invasion (P=0.0469), and peritoneal metastasis (P=0.0458). The rates of poor or undifferentiated tumours and peritoneal metastasis in the group of GA patients with a high density of IM-TLSs with GCs were 9.1% and 13.9%, respectively, which were much lower than those in the low-density group of GA patients (90.9% and 72.7%, respectively).

**Table 2:** Association Between the Density of TLSs with GC located in IM and Clinicopathological Parameters in patients with GA from FUSCC (166 cases).

Variables	IM TLS with GC		P value
	Low (n = 44)	High (n = 122)	
Age			
<55	15	56	0.1746
≥55	29	66	
Gender			
Male	24	77	0.3181
Female	20	45	
Tumour Location			
Cardia, Proximal or Fundus	17	54	0.5179
Body, Antrum or Distal	27	68	
Tumour size			
< 4.7 cm	26	75	0.7812
≥ 4.7 cm	18	47	
Differentiation			
Good or moderate	4	111	0.0001*
Poor or undifferentiated	40	11	
Lauren classification			
Intestinal	5	27	0.5587
Diffuse	21	52	
mixed	16	37	
Vascular invasion			
Absent	6	21	0.5815
Present	38	101	
Nervous invasion			
Absent	4	25	0.1070
Present	40	97	
Tumour invasion depth			
T1 + 2	2	20	0.0469*
T3 + 4	42	102	
Lymphatic metastasis			
Absent	0	2	0.999
Present	44	120	
Peritoneal metastasis			
Absent	32	105	0.0458*
Present	12	17	
pTNM stage			
I+II	3	22	0.0746
III+IV	41	100	

*Significance level: p < 0.05. The bold values represent p<0.05.

In summary, our study revealed that the spatial arrangement and maturation of TLSs were significantly correlated with the prognosis of patients with GA. Moreover, the abundance of tumour TLSs was an effective predictor of a favourable prognosis in patients with GA. Furthermore, the proportion of IM-TLSs with GCs showed a stronger association with recurrence and metastasis risk than did the total TLS density. Our data also showed that a high density of IM-TLSs in patients with GC was positively associated with high tumour histologic grade, high tumour invasion depth, and peritoneal metastasis in patients with GA. Thus, IM-TLSs with GCs in patients with GA can be considered a crucial clinicopathological parameter and a strong prognostic indicator.

### IM-TLSs with GC-high tumours exhibit robust DC-LAMP+ cell and PD-1+CD20+ B-cell infiltration and weak FOXP3+CD8+ Treg cell infiltration

**Figure 3:**
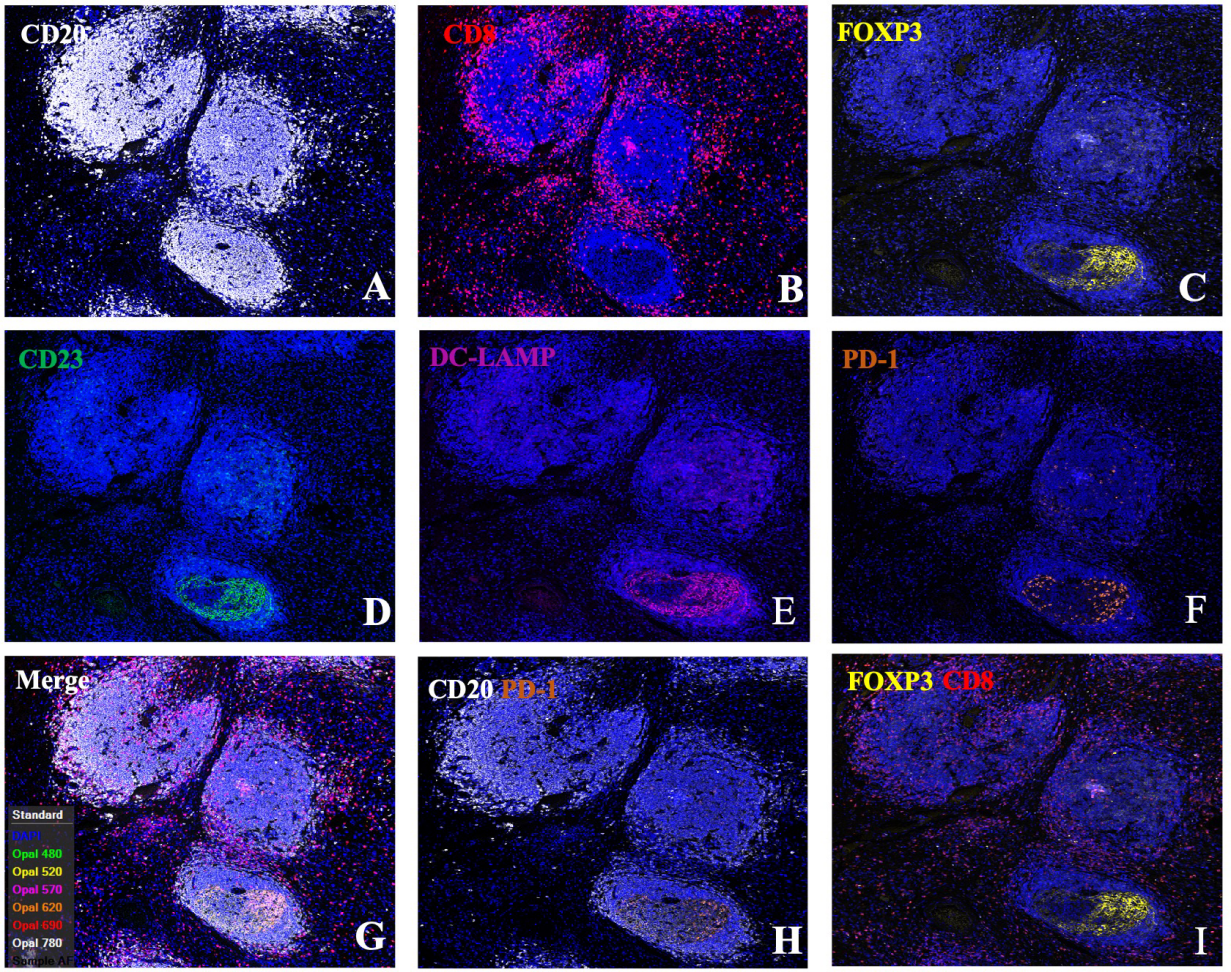
Single-channel and dual-channel images of mIF staining in tertiary lymphoid structures. Images of TLSs showing CD20 **(A)**, CD8 **(B)**, FOXP3 **(C)**, CD23 **(D)**, DC-LAMP **(E)**, and PD-1 **(F)** expression; merged images **(G)**; and CD20+PD1+ **(H)** and FOXP3+CD8+ cells **(I)**. FFPE sections of GC tissues were stained for CD20, CD8, FOXP3, CD23, DC-LAMP, and PD-1. Images were acquired using virtual microscopy and a Pannoramic scanner (Pannoramic MIDI, 3DHISTECH Ltd, Hungary).

**Figure 4:**
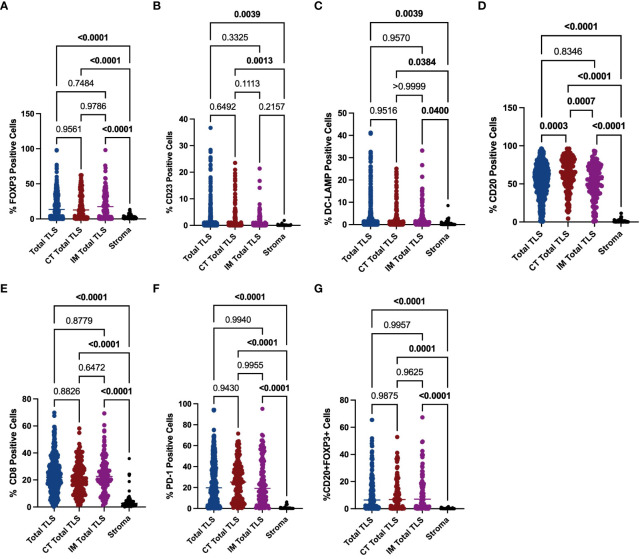
Evaluation of FOXP3, CD23, DC-LAMP, CD20, CD8 and PD-1 expression in tertiary lymphoid structures and the stroma. The scatter plot shows that the percentages of Foxp3+ Treg cells **(A)**, CD23+ GC B cells **(B)**, DC-LAMP+ mature DCs **(C)**, CD20+ B cells **(D)**, CD8+ T cells **(E)** and PD-1+ cells **(F)** and CD20^+^ Foxp3^+^ cells **(G)** were significantly greater in TLSs than in the tumour stroma in the indicated tissue samples. Moreover, the percentage of CD20+ B cells located in CT-TLSs was greater than that in either IM-TLSs or total TLSs **(D)**. Data comparisons among the groups were performed using one-way ANOVA.

To explore the mechanisms underlying the prognostic value of TLSs in the immune microenvironment of GA, we investigated the components of TLSs. To this end, we developed a 6-colour mIF panel that enabled simultaneous detection of CD8+ T cells, CD20+ B cells, CD23+ GC B cells, DC-LAMP+ mature DCs, PD-1+ cells, CD20+FOXP3+ cells, and Foxp3+ Tregs. The panel was applied to whole sections of 12 GA tumours. Six prominent immune subsets were identified by mIF staining (**Figure 3**). The distributions of these subsets were significantly different between TLSs and the tumour stroma. The percentages of Foxp3+ Treg cells, CD23+ GC B cells, DC-LAMP+ mature DCs, CD20+ B cells, CD8+ T cells, PD-1+ cells and CD20+ FOXP3+ cells were significantly greater in TLSs than in the tumour stroma (**Figures 4A–G**). However, there were no significant differences in the distributions of other subsets, except for CD20+ B cells, between IM-TLSs and CT-TLSs. The percentage of CD20+ B cells located in CT-TLSs was greater than that in IM-TLSs or total TLSs (IM-TLSs, p<0.0007; CT-TLSs, p<0.0003; **Figure 4D**).

**Figure 5:**
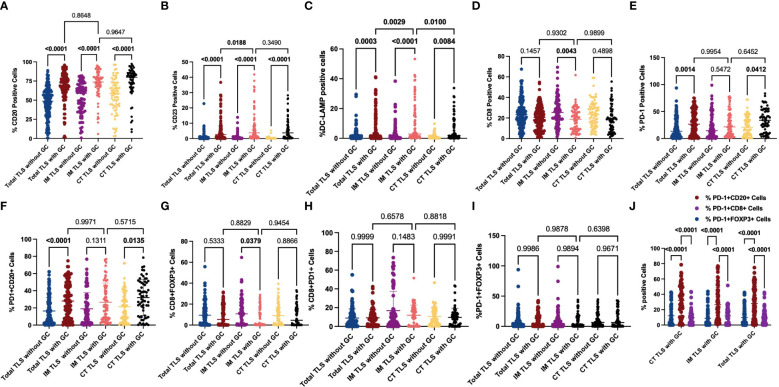
Evaluation of FOXP3, CD23, DC-LAMP, CD20, CD8 and PD-1 expression and possible coinfiltration patterns in tertiary lymphoid structures with or without GCs. **(A, B)** The scatter plot shows that the percentages of CD20+ **(A)** and CD23+ **(B)** cells were significantly greater in TLSs with GCs than in those without GCs. **(C)** The scatter plot shows that the percentage of DC-LAMP+ DCs was significantly greater in TLSs with GCs than in TLSs without GCs. Among the TLSs with GCs, the frequency of DC-LAMP+ DCs in IM-TLSs with GCs was significantly greater than that in either total TLSs or CT-TLSs with GCs. **(D)** The scatter plot shows that the percentage of CD8+ T cells in IM-TLSs decreased in parallel with the GC abundance in IM-TLSs. **(E)** The scatter plot shows that the percentage of PD-1+ cells increased in parallel with the GC abundance in total TLSs and CT-TLSs; **(F)** The scatter plot shows that the percentage of PD1+CD20+ B cells was greater in TLSs with GCs than in TLSs without GCs. **(G)** The scatter plot shows that the percentage of CD8+ FOXP3+ Treg cells was significantly lower in IM-TLSs with GCs than in IM-TLSs without GCs. **(H, I)** There was no difference in the proportion of PD-1+CD8+ T cells or PD-1+FOXP3+ Treg cells among TLSs with different distributions and maturity statuses. **(J)** The scatter plot shows that B cells in the GCs of TLSs, rather than CD8+ T cells or FOXP3+ Treg cells, highly expressed PD1.

To further explore the mechanisms underlying the prognostic value of IM-TLSs in patients with GC, we compared the differences in the above immune components among IM-TLSs, CT-TLSs, and total TLSs with or without GCs. We evaluated the presence of GCs in TLSs by staining for CD23 in serial sections (**Figure 3D**). First, the percentages of CD20+ (IM-TLSs with GCs: p<0.0001, CT-TLSs with GCs: p<0.0001, total TLSs with GCs: p<0.0001; **Figure 5A**) and CD23+ GC B cells (p<0.0001, p<0.0001, p<0.0001, **Figure 5B**) and DC-LAMP+ mature DCs (p=0.0003, p<0.0001, p=0.0084, **Figure 5C**) were significantly greater in TLSs with GCs, including IM-TLSs with GCs, CT-TLSs with GCs and total TLSs with GCs, compared with the corresponding tumour TLSs without GCs. Among TLSs with GCs, the frequency of DC-LAMP+ DCs in IM-TLSs with GCs was significantly greater than that in either total TLSs or CT-TLSs with GCs (p=0.0029, p=0.01; **Figure 5C**). The above results indicated that there is a potential correlation between the tumour immune activation milieu and the DC-LAMP+ DC abundance in IM-TLSs. Second, within IM-TLSs, the percentage of CD8+ T cells decreased in parallel with the GC abundance (p=0.0043, **Figure 5D**). Third, within total and CT-TLSs, the percentage of PD-1+ cells increased in parallel with the GC abundance (p=0.0014, p=0.0412, **Figure 5E**). Notably, compared with that in IM-TLSs without GCs, the percentage of PD-1+ cells was increased in IM-TLSs with GCs, although the difference was not statistically significant. The above results prompted us to focus more strongly on DC-LAMP+ DCs, CD8+ T cells, and PD-1+ cells.

To study the possible coinfiltration patterns of TLSs across different tumour regions, we performed hierarchical clustering of all acquired tumour and stromal tissues and TLSs with or without GCs, stratified by the frequencies of CD8+ PD1+ T cells, FOXP3+CD8+ T cells, PD-1+CD20+ B cells, and PD1+FOXP3+ Treg cells. We found that (1) these subsets predominantly infiltrated the stroma at a low frequency (p<0.0001, p<0.0001, p<0.0001; **Supplementary Figure 1**); (2) compared with that in TLSs without GCs, the percentage of PD1+CD20+ B cells was increased in TLSs with GCs (**Figure 5F**; CT-TLSs with GCs vs. CT-TLSs without GCs, p=0.0135; IM-TLSs with GCs vs. IM-TLSs without GCs, p=0.1311); (3) the percentage of CD8+ FOXP3+ Treg cells was significantly lower in IM-TLSs with GCs than in IM-TLSs without GCs (p=0.0379, **Figure 5G**); and (4) the proportions of PD1+CD8+ T cells and PD1+FOXP3+ Treg cells did not differ among TLSs with different distributions and maturity statuses (**Figures 5H, I**). Notably, B cells in the GCs of TLSs, rather than CD8+ T cells or FOXP3+ Treg cells, highly expressed PD1 (p<0.0001, **Figure 5J**). Taken together, these findings indicate that IM-TLSs with GCs in GA are associated with increased infiltration of DC-LAMP+ DCs and decreased infiltration of CD8+ Tregs. In addition, PD1 is highly expressed in B cells in TLSs with GCs.

### Gene signature enrichment analysis

**Figure 6:**
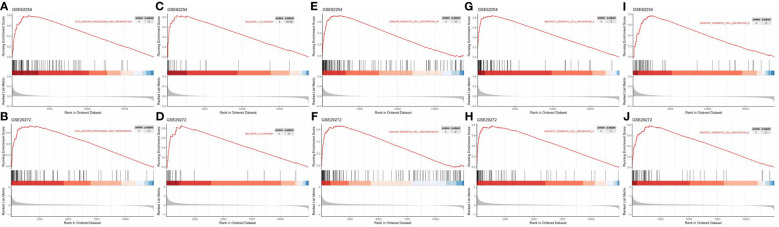
GSEA revealed enriched pathways involved in the function and maturation of DCs in the TLS+ group. **(A, B)** GSEA results showing the enrichment of pathways involved in antigen processing and presentation in both datasets; **(C, D)** GSEA results showing the enrichment of the IL12 pathway in both datasets. **(E–J)** GSEA results showing the enrichment of pathways related to DC maturation in both datasets.

To further explore the role of TLSs in antitumour immunity, we performed GSEA to compare the TLS+ group and TLS- group. In the GSE62254 dataset, 1490 pathways were enriched (p<0.05, q<0.25) in the TLS+ group compared with the TLS- group (**Supplementary Table 3**), and in the GSE29272 dataset, 1004 pathways were enriched in the TLS+ group (**Supplementary Table 4**). A total of 746 pathways were enriched in both datasets, among which many were immune-related pathways, including but not limited to gene sets involved in DC maturation, antigen processing and presentation, and the IL12 pathway (**Figure 6**).

## Discussion

The molecular subtyping of cancer plays an important role in predicting tumour outcomes and guiding tumour treatment. However, to date, molecular subtyping has not replaced the traditional method for classification of cancer, which is performed by pathologists based on histological appearance. In contrast, the evaluation of pathological parameters based on tumour histological morphology has recently become a focus because this method is easy to perform and imposes only a small financial burden on medical systems. For instance, tumour budding is defined as the presence of single cells or clusters of up to four cells at the IM of CRC tumours (30), and it has been proven to be a well-established independent prognostic factor for lymph node metastasis and survival (31). Similarly, TLSs could also be an element for evaluation in the clinical setting. Although TLSs have specific morphological characteristics and are relatively small in size, their functions in the local microenvironment are important for both cellular and humoral immune responses directed against neoplastic cells, and they have been demonstrated to be a prognostic factor in many solid tumours and to be a predictor of treatment efficacy in melanoma and lung cancer (32–34).

To evaluate whether TLSs could serve as prognostic indicators in GA, as observed for other solid tumours, we used public RNA-seq data to calculate the TLS score using published gene signatures. Notably, TLSs were found to be a favourable prognostic factor in patients with GA. Next, an evaluation of the TIME was performed, showing that the TLS+ group had higher scores for the GCs and almost every type of immune cell, including activated DCs. Since GCs are frequently observed in TLSs and studies have shown that GCs are associated with a longer survival time in other types of cancers (24, 26), we sought to confirm this finding in gastric cancer at the transcriptome level. The TLS+GC+ group had a better prognosis than the TLS+GC- group, while the TLS-GC+ group did not have a better prognosis than the TLS-GC- group, indicating that GCs are likely to be a favourable prognostic factor in TLS+ GA. We also found that the DC scores were positively correlated with the GC scores in the GA database. The TLS+GC+DC+ group had a longer survival time than the TLS+GC+DC- group, indicating that DCs are likely to be a favourable prognostic factor in TLS+GC+ GA. However, traditional bulk RNA-seq analysis reflects gene expression at the transcriptional level in tumours and does not consider the morphology and location of TLSs; thus, further validation is needed in real-world tumour specimens.

By analysis of the FUSCC GA cohort, we revealed the complex role of TLSs based on their density and spatial location in patients with GA. We found that high densities of total TLSs and total IM-TLSs were significantly correlated with prolonged OS, while total CT TLSs were not correlated with prognosis. We also assessed the maturation stages of TLSs and revealed that the abundance of IM-TLSs with GCs was an effective predictor of favourable OS and DFS in patients with GA. In addition, the density of IM-TLSs with GCs in the FUSCC GA cohort was correlated with histologic grade, depth of tumour invasion and peritoneal metastasis status. The density of IM-TLSs with GCs and the peritoneal metastasis status were independently associated with OS and DFS. As found in the current study and previous studies, TLSs with different spatial locations and maturity statuses have different functions (5, 15). Li et al. reported that compared with intratumoural TLSs, peritumoural TLSs were associated with a lower risk of cancer recurrence and more favourable outcomes in patients with HCC (35). He et al. also reported that IM-TLSs were associated with a longer OS time in patients with GA (36). Moreover, Posch. et al. reported that the presence of at least one GC-harbouring TLS was associated with a much lower risk of CRC recurrence (15). These authors proposed that TLSs with a GC reaction represent the most “functional” subtype of TLSs and that the presence of GC+ TLSs also reflects an immunogenic TME. Therefore, not only the number but also the distribution and maturation dynamics of TLSs provide prognostic information, yet the underlying mechanism remains unclear.

Herein, we found that compared to that in tumour TLSs without GCs, the frequency of DC-LAMP+ mature DCs was significantly greater in TLSs with GCs. The frequency of DC-LAMP+ mature DCs in IM-TLSs with GCs was significantly greater than that in total TLSs or CT-TLSs with GCs. The above results indicated that there is a potential correlation between the tumour immune activation milieu and the abundance of DCs in IM-TLSs. DCs are specialised antigen-presenting cells that can be found in the paracortical area of the lymph nodes, where they promote the activation of naive T lymphocytes. Studies have shown that CD83+ DCs (equivalent to DC-LAMP+ DCs) are distributed predominantly in the IM of the tumour stroma and are tightly attached to B-cell lymphoid follicles resembling GCs (37). Furthermore, in primary melanoma, a high density of DC-LAMP+ mature DCs within lymphoid aggregates was found to be associated with strong infiltration of activated T cells and a significantly increased DFS rate (38). Therefore, we speculated that IM-TLSs containing DC-LAMP+ mature DCs were released in a mature and activating state and could promote the antitumour immune functions of IM-TLSs and, moreover, that the high abundance of mature DCs within TLSs may constitute the basis for the favourable prognostic impact. Our GSEA revealed that a low TLS score was associated with a relatively “cold” TIME, while a high TLS score indicated a more active TIME. We found the enrichment of a notable number of DC-related functions and pathways in the TLS+ group. DCs promote immunity by presenting antigens to T cells through cell−cell contacts and cytokines and play a central role in antigen-specific immunity (39). We found that pathways including but not limited to antigen processing and presentation, the IL-12 pathway, and DC maturation were highly enriched in the TLS+ group, suggesting that DCs likely contribute to the favourable prognostic impact of TLSs.

Moreover, within IM-TLSs, the percentage of CD8+ T cells decreased in parallel with the GC abundance. The IM is generally considered the frontline of the tumour-host interaction. Previous results indicate that the CD8+ cell density in the IM correlates with a favourable clinical outcome in CRC (40), whereas the opposite conclusion was reached for breast cancer (41); however, currently, it is not known whether the opposite predictive direction is due to migratory processes induced by locally secreted factors or architectural barriers capturing these cells in the tumour compartments or because functional CD8+ T cells account for only a small portion of the total CD8+ T cell population.

We also noted significant enrichment of PD-1+ cells in TLSs with GCs compared with that in the counterpart TLSs without GCs. Programmed death-1 (PD-1) is reported to be expressed mainly on functionally “exhausted” CD8+ T cells, dampening the host antitumour immune response (42). We found that B cells in the GCs of TLSs, rather than CD8+ T cells or FOXP3+ Treg cells, highly expressed PD1. PD-1 is involved in the negative regulation of T-cell activation. The role of PD-1 in T cell activation is well established, but its role in B cell activation has not been well studied. Previous studies have shown that B cells are a predictor of the response to anti-PD-1 inhibitor therapy in several types of cancer (43). PD-1 expressed in B cells is also a target of anti-PD-1 blockade therapy. Disruption of the PD-1-PD-L1 interaction increases B-cell activation, proliferation and cytokine production, which might be why anti-PD-1/PD-L1 inhibitors can exhibit effectiveness in some tumours with few PD-1+ T cells (44). In immature TLSs, B cells might acquire immunosuppressive functions, whereas B cells in mature TLSs can undergo maturation, selection, amplification, somatic hypermutation and affinity maturation, and immunoglobulin class switching, leading to the production of plasma cells that secrete IgG or IgA (45). Therefore, we speculate that plasma cells generated *in situ* within mature TLSs can produce antibodies that target specific tumour-associated antigens.

Overall, our findings indicate that the distinct cellular composition of TLSs within and around tumours may determine their pro- or antitumour effects. We also found that compared to that in TLSs without GCs, the proportion of FOXP3+CD8+ Treg cells was significantly lower in IM-TLSs with GCs, suggesting a potential connection between IM-TLSs and the intratumoural immunosuppressive milieu. As a potent regulatory population, CD8+Foxp3+ T cells have a transcriptional profile and suppressive function similar to those of CD4+ Tregs and distinct from those of conventional CD8+ T cells (46). CD8+Foxp3+ T cells have been reported to play a role in several pathological and physiological processes, including cell death, necrosis, and apoptosis; viral infection; and immune cell proliferation (46). CD8+FoxP3+ T cells were also previously described as CD8+ Tregs with immunosuppressive functions in tumours (47). CD8+Foxp3+ T cells may perform a unique function in regulating B-cell responses. For example, CD8+ Treg cells have been found to suppress B-cell proliferation and immunoglobulin production (48).

In summary, the work presented here extends the present knowledge in the following ways: (1) public RNA-seq datasets were analysed to evaluate the prognostic roles of TLSs, GCs and DCs; (2) a novel scoring system was established to quantify the abundance and evaluate the spatial distribution of TLSs; (3) a sequential process of maturation of TLSs in GA was discovered; (4) the prognostic value of TLSs and their correlations with clinicopathological parameters were assessed; and (5) the different cellular components of TLSs were identified, and their relationships with GCs were studied. However, the primary limitation of our study is that it was a single-centre retrospective study with a relatively small sample size, supplemented by analysis of public bulk RNA-seq data. Validation of our results in prospective and larger cohort studies would be beneficial. In addition, traditional bulk RNA-seq analysis reflects the gene expression level in the CT, neglecting the potential for the IM of the tumour to convey more information since it is the frontline of the tumour-host interaction. However, by evaluation of H&E-stained sections, the spatial variance could be assessed accurately and analysed. Furthermore, scRNA-seq technology has advanced greatly in recent years, and combining this approach with bench studies will certainly improve the comprehensiveness of future research. In addition, to our knowledge, this is the first report regarding the study of the maturation stage of TLSs and how TLS maturity affects prognosis in patients with GA. Our findings provide a basis for future investigations on the formation and development of TLSs in GA and for the development of possible precision therapies exploiting TLSs.

## Data availability statement

The raw data supporting the conclusions of this article will be made available by the authors, without undue reservation.

## Ethics statement

The studies involving humans were approved by The Research Ethics Committee of FUSCC. The studies were conducted in accordance with the local legislation and institutional requirements. The human samples used in this study were acquired from primarily isolated as part of your previous study for which ethical approval was obtained. Written informed consent for participation was not required from the participants or the participants’ legal guardians/next of kin in accordance with the national legislation and institutional requirements.

## Author contributions

HS: Writing – original draft, Formal analysis, Data curation. YL: Writing – original draft. WC: Writing – original draft. RX: Writing – original draft, Data curation. WG: Writing – original draft, Formal analysis. XZ: Writing – original draft, Methodology. XiW: Writing – original draft, Data curation. XuW: Writing – original draft, Formal analysis. CT: Writing – original draft, Project administration. WW: Writing – original draft, Investigation. MZ: Writing – original draft, Methodology. SN: Writing – original draft, Formal analysis, Data curation. DH: Writing – original draft, Data curation. MX: Writing – original draft, Project administration. WS: Writing – review & editing. LW: Writing – review & editing.
