# Supplementary material for: The distribution and maturation of tertiary lymphoid structures can predict clinical outcomes of patients with gastric adenocarcinoma
Source: Front Immunol. 2024 Jul 29;15:1396808. doi: 10.3389/fimmu.2024.1396808 (PMC11317265; doi:10.3389/fimmu.2024.1396808)
Supplement: Supplementary file 2 [file Table_2.docx]

**Supporting Information-Table S2.** The optimal cutoff values for TLS density were determined using the maxstat package of R.

| Variable | Cut-off(/100mm^2^) | |  |
| --- | --- | --- | --- |
|  | OS | DFS | |
| CT Total TLS | 16.9 | 6.02 | |
| CT TLS with GC | 3.81 | 2.92 | |
| CT TLS with HEV | 0.18 | 2.59 | |
| CT TLS with HEV and GC | 0.99 | 0.17 | |
| IM Total TLS | 6.69 | 12.70 | |
| IM TLS with GC | 0.31 | 0.31 | |
| IM TLS with HEV | 0.67 | 2.08 | |
| IM TLS with HEV and GC | 0.34 | 0.00 | |
| Total TLS | 15.64 | 10.45 | |
| Total TLS with GC | 0.93 | 0.45 | |
| Total TLS with HEV | 1.47 | 7.60 | |
| Total TLS with HEV and GC | 0.48 | 1.40 | |
