# Supplementary figures and images for: The distribution and maturation of tertiary lymphoid structures can predict clinical outcomes of patients with gastric adenocarcinoma
Source: Front Immunol. 2024 Jul 29;15:1396808. doi: 10.3389/fimmu.2024.1396808 (PMC11317265; doi:10.3389/fimmu.2024.1396808)

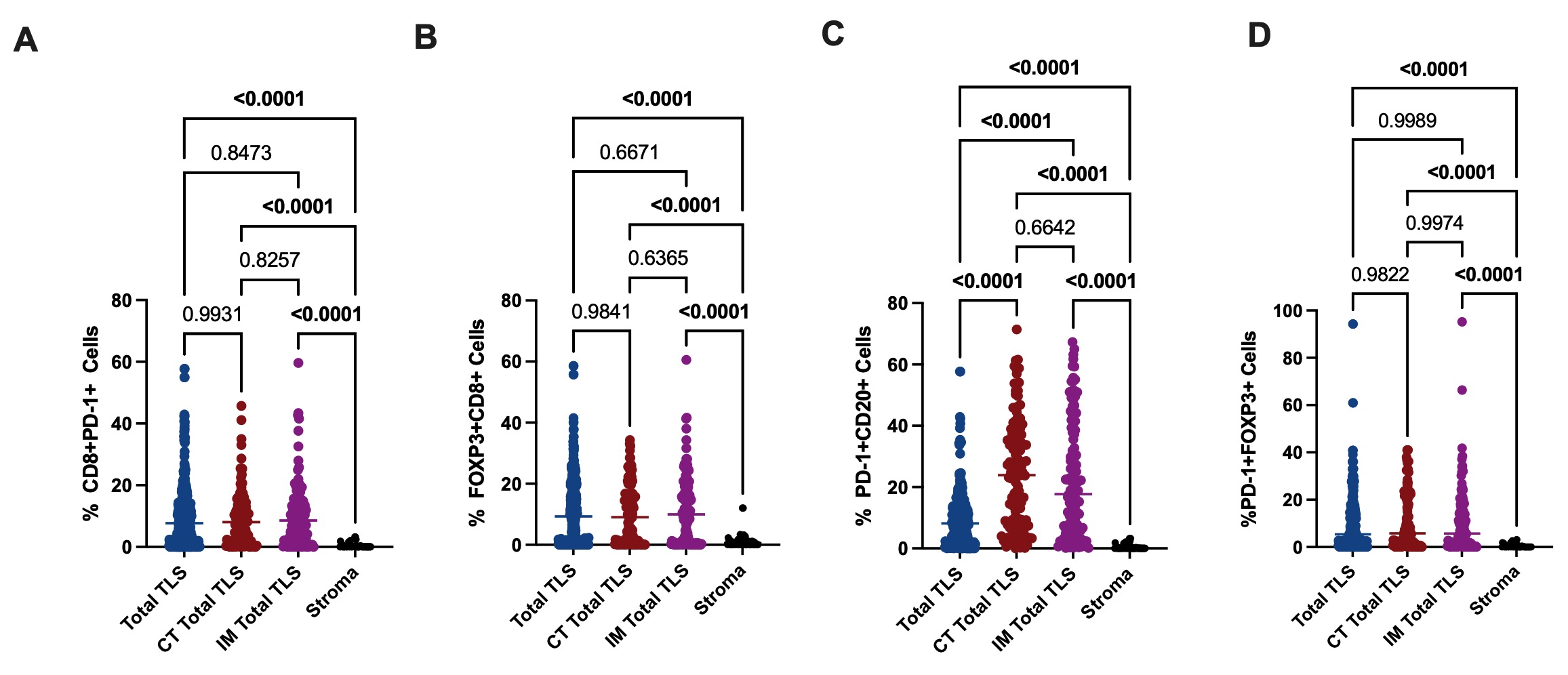

Supplement: Supplementary file 5 [file Image_1.jpeg]
